# Dual-functional quantum-dots light emitting diodes based on solution processable vanadium oxide hole injection layer

**DOI:** 10.1038/s41598-021-81480-5

**Published:** 2021-01-18

**Authors:** Tae Yeon Kim, Sungho Park, Byung Jun Kim, Su Been Heo, Jong Hun Yu, Jae Seung Shin, Jong-Am Hong, Beom-Su Kim, Young Duck Kim, Yongsup Park, Seong Jun Kang

**Affiliations:** 1 Department of Advanced Materials Engineering for Information and Electronics (BK21 four), Kyung Hee University, Yongin, 17104 Korea; 2 Department of Physics and Research Institute for Basic Sciences, Kyung Hee University, Seoul, 02447 Korea

**Keywords:** Optoelectronic devices and components, Electronic devices

## Abstract

Dual-functional quantum-dots light emitting diodes (QLEDs) have been fabricated using solution processable vanadium oxide (V_2_O_5_) hole injection layer to control the carrier transport behavior. The device shows selectable functionalities of photo-detecting and light-emitting behaviors according to the different operating voltage conditions. The device emitted a bright green light at the wavelength of 536 nm, and with the maximum luminance of 31,668 cd/m^2^ in a forward bias of 8.6 V. Meanwhile, the device could operate as a photodetector in a reverse bias condition. The device was perfectly turned off in a reverse bias, while an increase of photocurrent was observed during the illumination of 520 nm wavelength light on the device. The interfacial electronic structure of the device prepared with different concentration V_2_O_5_ solution was measured in detail using x-ray and ultraviolet photoelectron spectroscopy. Both the highest occupied molecular orbital and the gap state levels were moved closer to the Fermi level, according to increase the concentration of V_2_O_5_ solution. The change of gap state position enables to fabricate a dual-functional QLEDs. Therefore, the device could operate both as a photodetector and as a light-emitting diode with different applied bias. The result suggests that QLEDs can be used as a photosensor and as a light-emitting diode for the future display industry.

## Introduction

Photosensor has been considered as a key component of optoelectronics for the next-generation smart device according to the recent development of internet of thing (IoT) technology^1,2^. Various photosensors could be integrated into IoT circuits to detect a light including infrared, visible, ultraviolet light, and to convert the input light signal into the electrical signal^3–5^. Based on the detection of light, IoT circuits can observe the environmental condition and perform many useful functions, such as an autonomous car, a smart factory, and the next-generation communications^6–8^. Therefore, there are many research efforts to develop a high-performance photosensor based on emerging nanomaterials^9,10^.

Recently, detecting visible-light is considered as the most important function for the IoT photosensor, because of the similarity with human eye and development of artificial intelligence^11,12^. Also, many research groups tried to demonstrate unconventional visible-light photosensors, such as a highly-transparent and multi-functional visible-light photosensors, due to their flexibility for the application in IoT fields^13–15^. Recently, a visible-light photosensor based on a hetero-structure of small band gap quantum-dots and wide band gap oxide semiconductor has been developed^16,17^. A visible-light photosensor base on 2D-nanomaterials have been demonstrated recently by Xiao et al., as well^18^. Although most of the image sensors are composed of photodiodes, recent developments have demonstrated a visible-light phototransistor. Therefore, it is necessary to develop a photodiode, which can detect a visible-light, for the next-generation IoT photosensor.

Meanwhile, quantum-dots light-emitting diodes (QLEDs) have been considered as a next-generation display due to its superior electrical and optical properties than the organic light-emitting diodes^19,20^. Colloidal quantum-dots (QDs) are widely used in optoelectronics due to their high stability and durability^21^. Beside, QDs have a tunable band gap by controlling the size of QDs, excellent color purity, and high external quantum efficiency^22,23^. Most of all, QLEDs can be fabricated using a solution process which is favorable for the large-scale display with a low cost in industry. Therefore, QLEDs can be applied to the thin and deformable display with a high-performance for the IoT optoelectronics. Basically, QLEDs and QDs photodiode have a same device structure. The difference of QLEDs and QDs photodiode is the operating condition. Generally, a positive bias is applied for the light-emitting state of QLEDs, while a negative bias is applied for the light-detecting of QDs photodiode^24^. Therefore, dual-functional optoelectronics, which can emit and detect a visible-light, can be fabricated using QDs and allow the developments of smaller and high-performance IoT optoelectronics. However, it is hard to achieve an efficient interfacial electronic structure for the high efficient light emission and photodetection. Therefore, it is important to control the interfacial electronic structure of the device to achieve a dual functional QLEDs.

In this study, we have fabricated and evaluated dual-functional QLEDs that used a solution processable inorganic V_2_O_5_ hole injection layer (HIL). The change of interfacial electronic structure by inserting various concentrations of V_2_O_5_ has been measured using x-ray photoelectron spectroscopy (XPS) and ultraviolet photoelectron spectroscopy (UPS). Based on the interfacial electronic structure, we have optimized dual-functional QLEDs to have a light-emitting and a photo-detecting abilities in a visible range. Time-resolved photoluminescence (TRPL) measurements were performed to confirm the photon-electron energy transfer mechanism. In addition, circuits that can simultaneously generate and exchange electrical signal to optical signal, and vice versa were demonstrated using two dual-functional QLEDs. Therefore, the device can be applicable as a next-generation IoT optoelectronics.

## Results and discussion

**Figure 1:**
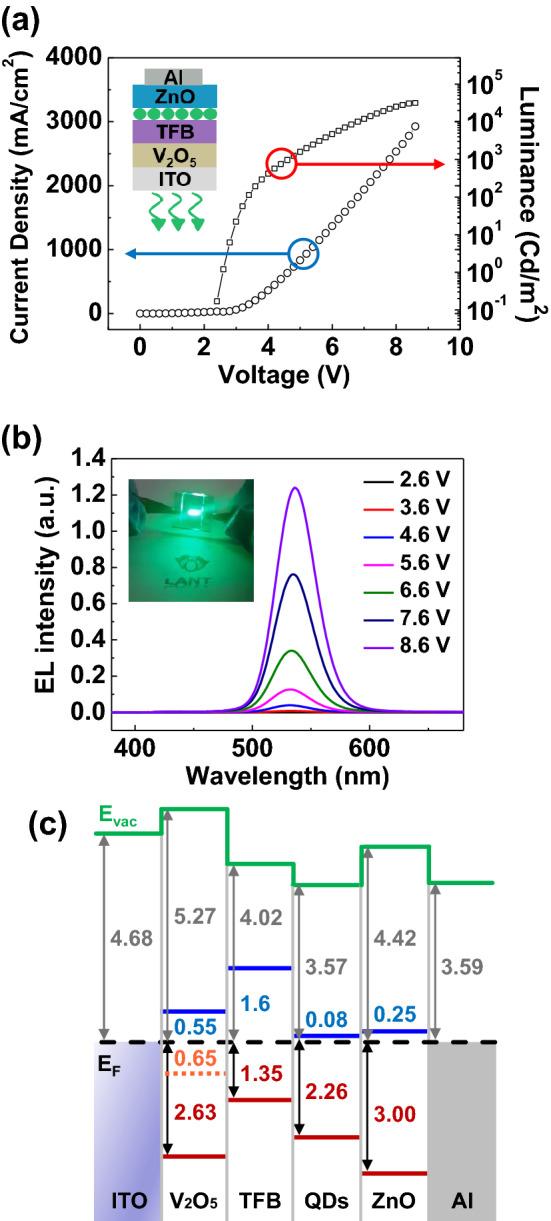
(**a**) Current density and luminance versus applied voltage curves of QLEDs. (J–L–V) Inset shows a schematic illustration of the QLEDs under forward bias for the light emission. (**b**) Electroluminescence spectra of QLEDs. Inset shows the light emitting QLEDs. (**c**) Energy level diagram of the QLEDs measured from UPS. The concentration of V_2_O_5_ was 1 wt%.

In this work, conventional structured QLEDs have been fabricated using a solution processable V_2_O_5_ HIL, and characterized the electrical and optical properties of QLEDs as shown in Fig. 1. The device consists of thin multilayers of ITO/V_2_O_5_/TFB/QDs/ZnO/Al, schematic of fabricated QLED is shown in the inset of Fig. 1a. Core/shell structured CdSe/ZnS QDs were used as an emissive layer, while TFB and ZnO are used as a hole transport layer (HTL) and electron transport layer (ETL), respectively. 1 wt% of V_2_O_5_ solution has been used to form a thin layer of HIL for the conventional structured QLEDs. Current density and luminance characteristics according to the applied voltage are shown in Fig. 1a. The turn-on voltage was 2.6 V, and the device exhibited the maximum luminance of 31,668 cd/m^2^ at 8.6 V. Figure 1b shows the electroluminescence (EL) spectra of the QLEDs according to increase the applied forward bias on the device. The peak position of the EL spectra was 536 nm regardless of the applied voltage. The full width at half maximum (FWHM) was 39 nm, which is narrow enough to exhibit excellent color purity. The image of bright green light emitting QLEDs is shown in the inset of Fig. 1b. The interfacial electronic structure of QLEDs was measured using UPS, and energy level diagram is shown in Fig. 1c. The work functions of ITO and Al, and the valence band maximum (VBM) of V_2_O_5_, TFB, QDs, and ZnO were measured by preparing multilayer films of ITO, ITO/V_2_O_5_, ITO/V_2_O_5_/TFB, ITO/V_2_O_5_/TFB/QDs, ITO/V_2_O_5_/TFB/QDs/ZnO, and ITO/V_2_O_5_/TFB/QDs/ZnO/Al, respectively. The secondary electron cutoff (SEC) and the highest occupied energy state molecular orbital (HOMO) values of each thin films was obtained from the spectra as shown in Figure S1. The work functions of ITO and Al were measured as 4.68 eV and 3.59 eV, respectively. The VBM of V_2_O_5_ (1 wt%) was measured as 2.63 eV below the Fermi energy (E_F_), while the distance between the vacuum level (E_V_) and the E_F_ was obtained as 5.27 eV. As shown in the inset of Figure S1, the gap state was observed 0.65 eV below the E_F_ in V_2_O_5_ HIL. The gap state can promote the injection of hole carriers form ITO into TFB^25^. The optical band gap and the conduction band minimum (CBM) of V_2_O_5_ HIL were calculated from the Tauc’s plots of absorbance spectrum of V_2_O_5_ as shown in Figure S2. The CBM of V_2_O_5_ HIL was located 0.55 eV above the E_F_. The VBM and the CBM of TFB, QDs, and ZnO were obtained using the same process and summarized in the Fig. 1c. Through the gap state of V_2_O_5_ and the CBM of ZnO, hole and electron carriers can inject well into the emissive layer of QDs at the forward bias condition to emit a bright light.

**Figure 2:**
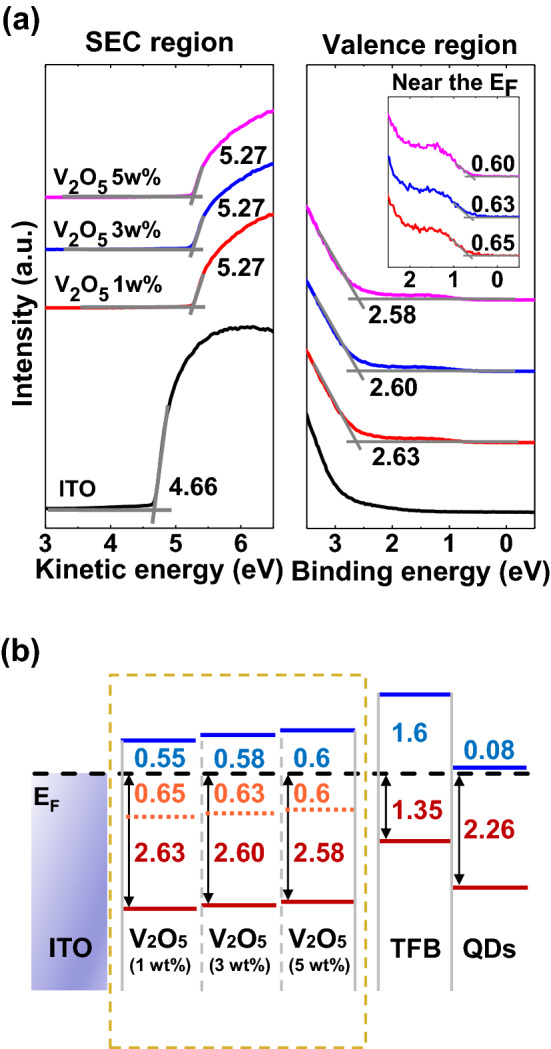
(**a**) UPS spectra of ITO and various concentration V_2_O_5_ films on ITO at the secondary cutoff and E_F_ regions. To clarify the gap state, inset shows near the E_F_. (**b**) Energy level diagram of the hole injection region of the QLEDs. Inside the dotted square, gap states and HOMO levels of different concentration V_2_O_5_ films on ITO are shown.

A bright light could be emitted from the QLEDs with an appropriate interfacial electronic structure for the efficient charge injection and transport. However, the off-current of the QLEDs is too high in reverse bias to be used as a photodetector. The thickness of ~ 1 wt% V_2_O_5_ HIL was ~ 6 nm^26^. Also the thickness of 3 wt% V_2_O_5_ and 5 wt% V_2_O_5_ was estimated using the cross-sectional TEM images as shown in Figure S3. The thickness of 3 wt% V_2_O_5_ was ~ 14.4 nm, while the thickness of 5 wt% V_2_O_5_ was ~ 25.9 nm. Therefore, the thickness of V_2_O_5_ HIL depends on the concentration of the vanadium precursor, and affects to the charge transfer. The thickness of the V_2_O_5_ HIL was controlled to increase the photoresponsivity by lowering the leakage current. QLEDs fabricated with the higher concentration (5 wt%) of V_2_O_5_ solution exhibits less currents at the reverse bias than the device fabricated with the lower concentration of V_2_O_5_ solution, as shown in Figure S4. Therefore, the device can be optimized to be applicable for a photosensor by controlling the V_2_O_5_ HIL and reducing the off current at the reverse bias. The interfacial electronic structures of ITO/V_2_O_5_ (1, 3, 5 wt%)/TFB/QDs were measured using UPS. Figure 2a shows the UPS spectra of bare ITO glass, V_2_O_5_ on ITO glass spin-coated with different concentrations of V_2_O_5_ solution. The work function of ITO was 4.66 eV, which can be obtained from the SEC region spectrum. According to increase the thickness of V_2_O_5_ HIL, the HOMO level shifts from 2.63 to 2.58 eV, and gap state energy level shifts from 0.65 to 0.6 eV from the Fermi level, while the density of state (DOS) of the V_2_O_5_ thin film depends on the precursor concentration as shown in the HOMO region and near the E_F_ (Inset of Fig. 2a). Since the gap state plays an important role in the hole injection and transport through the V_2_O_5_ HIL, the shift of gap state energy level is an important factor in the hole transfer mechanism. Figure 2b summarizes the SEC region and valence region spectra in Fig. 2a, and shows the interfacial energy level according to the different concentration of V_2_O_5_. The HOMO and gap state levels of the V_2_O_5_ HIL get closer to the E_F_ according to increase the concentration of V_2_O_5_ solution. In QLEDs mode, 0.7 eV offset of V_2_O_5_ (1 wt%) is a low offset energy barrier from V_2_O_5_ to TFB compared to the 0.75 eV offset of V_2_O_5_ (5 wt%), making hole relatively easily injected. The low offset between the gap state level of HIL and the HOMO level of HTL can improve the EL performance. The V_2_O_5_ (1 wt%) HIL showed the best EL characteristics due to the improvement of charge balance. The V_2_O_5_ (5 wt%) induces hole accumulation due to the high energy barrier, which causes a weakening of the current injection. Figure S5 shows the EL performances of the device with different vanadium concentrations. The 3 wt% and 5 wt% V_2_O_5_ QLEDs exhibited maximum luminance of 18,110 cd/m^2^ and 14,127 cd/m^2^, respectively. As the vanadium precursor concentration increase, the current density decrease. The EL specta have the peak position at 536 nm. Compared to the current efficiency for each concentration of V_2_O_5_, the maximum current efficiency of 1 wt% V_2_O_5_ is 1.09 cd/A. In PD mode, hole that is easily moved to high energy state are transferred regardless of the gap state of V_2_O_5_ depending on the concentration. However, V_2_O_5_ (1 wt%) indicates a leakage current that is too large compared to the generated photocurrent, so that no photoresponsivity occurs. On the other hand, the V_2_O_5_ (5 wt%) HIL decreases the leakage current as the thickness of HIL increases, to improve the photodetector.

**Figure 3:**
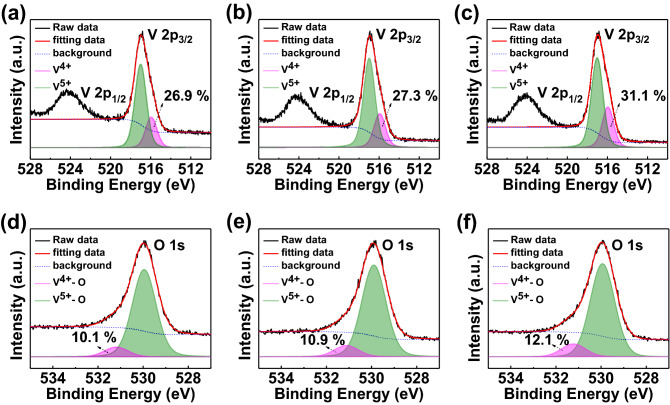
Measured V 2*p*_3/2_ and O 1*s* core level spectra of (**a**), (**d**) 1 wt% V_2_O_5_ film on ITO, (**b**), (**e**) 3 wt% V_2_O_5_ film on ITO, and (**c**), (**f**) 5 wt% V_2_O_5_ film on ITO. In the V 2*p* core-level spectra, the ratio of V^4+^ and V^5+^ are shown, while the ratio of oxygen binding ratio with V^4+^ and V^5+^ is shown in O 1*s* core-level spectra.

XPS measurements have been performed on each V_2_O_5_ thin film, fabricated with different concentration of the solution, to analyze the origin of the different DOS of the gap state energy level. Figure 3 shows the XPS core level spectra of vanadium (V 2*p*) and oxygen (O 1* s*) to analyze the composition of the V_2_O_5_ HIL. The peak positions for V 2*p*_1/2_, V 2*p*_3/2_, and O 1* s* were 524.3, 516.9 and 530 eV, respectively. There was no significant difference in the V 2*p*_1/2_ peak. However, asymmetric peak was observed in the low binding energy portion of the V 2*p*_3/2_ peak, which indicates the presence of various oxidation states of V_2_O_5_. The spectra of V 2*p*_3/2_ oxidation state can be separated to V^4+^ (515.9 eV) and V^5+^ (517 eV) through the Lorentzian-Gaussian fitting, as shown in Fig. 3a–c. The V^5+^ state at the higher binding energy indicates V_2_O_5_, while the V^4+^ state corresponds to VO_2_, which indicates the gap state. The amount of VO_2_ increases from 26.9 to 31.1% according to increase the concentration of V_2_O_5_ solution from 1 to 5 wt%. Therefore, it can be suggested that rich V^4+^ states of V_2_O_5_ shows a gap state closer to E_F_ based on the UPS and XPS measurements. Besides, the O 1*s* peak can be separated by V^4+^ (531.2 eV) and V^5+^ (530 eV) by Lorentzian-Gaussian fitting, as well, as shown in Fig. 3d–f. The amount of V^4+^ state increases from 10.1 to 12.1% according to increase the concentration of V_2_O_5_ solution from 1 to 5 wt%. Therefore, the result indicates that VO_2_ leads the increase of DOS of the gap state, and affects to the hole transfer behavior.

**Figure 4:**
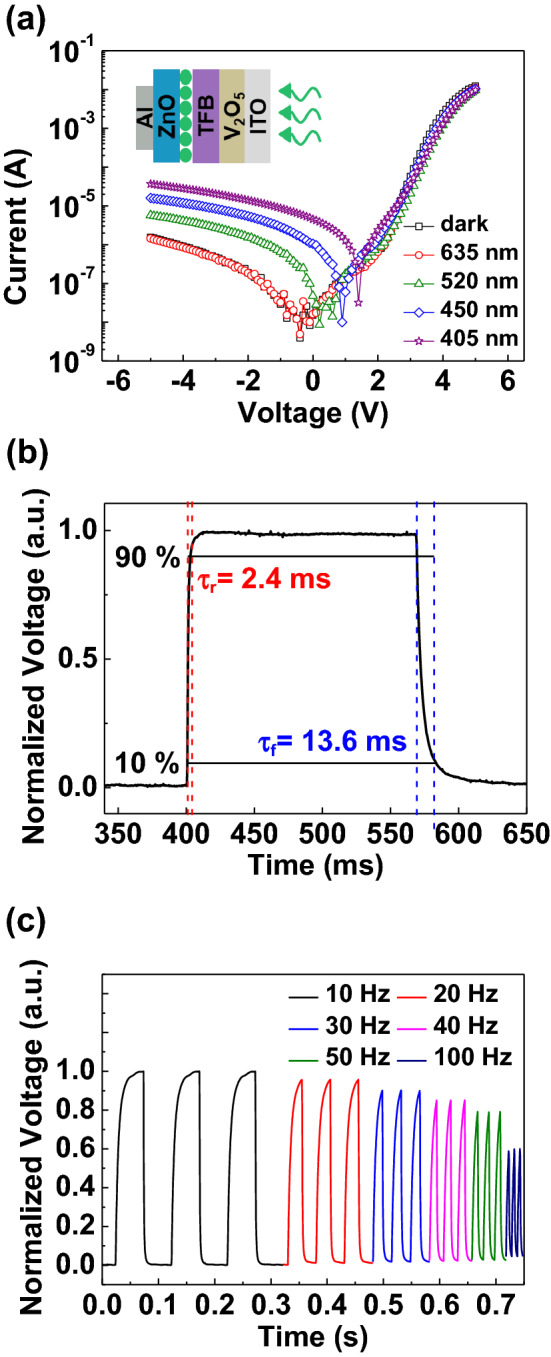
(**a**) Transfer curves of the device in a dark state and with the illumination of various wavelengths light. Inset shows a schematic illustration of the QLEDs with 5 wt% V_2_O_5_ for the light detecting process. (**b**) Photo response characteristics of the device. Rising and falling time was defined as the interval between 10 and 90% of the signal. (**c**) Normalized photo responses according to the different speed of turn on and off the 520 nm wavelength light signal.

To evaluate the photodetection behavior of the dual-functional QLEDs, a device was prepared with the 5 wt% V_2_O_5_ solution as shown in the inset of the Fig. 4a. Figure 4a shows the I–V characteristics of the dual-functional QLEDs with and without the illumination of light (λ = 405, 450, 520, 635 nm). Since the band gap of QDs was 2.34 eV, it is hard to observe a photocurrent with the illumination of 635 nm wavelength light (1.95 eV). Meanwhile, an increase of the photocurrent could be observed with the illumination of light (λ = 405, 450, 520 nm) on the device at the reverse bias. The photon energy of the light (λ = 405, 450, 520 nm) was high enough to excite carriers from the VBM to CBM of QDs. The device shows excellent photoresponsivity in light illumination over 520 nm (2.38 eV), because of band gap of the active layer CdSe/ZnS QDs, which has an absorbance peak around 520 nm, as shown in Figure S6. As the reverse bias voltage increases, the increase of the photocurrent was observed due to the strong external electric field to collect the charges. The photoresponsivity of dual-functional QLEDs at the photodetection mode can be calculated using the following equation.

$$\mathrm{R}= \frac{{I}_{light}- {I}_{dark}}{PA}$$

where *I*_*light*_ is the photocurrent under various wavelengths of illumination, *I*_*dark*_ is the dark current, *P* is the incident light power and *A* is the illuminated area of the device. The photoresponsivity was calculated and shown in Table S1. In our previously study, we reported a photodiode suing QDs and oxide semiconductor with the photoresponsivity of 0.09 A/W^16^. In this study, the dual-functional devices with V_2_O_5_ HIL have a photoresponsivity of 0.0076 A/W, which is similar to our previous study. Higher reverse bias improved the generation of the photo-excited carriers. The photoresponses of the dual-functional QLEDs with the periodic light illumination of various wavelengths of light and reverse biases are shown in Figure S7. Figure 4b shows a normalized, single rising and falling cycle measured with a 520 nm light illumination with − 2 V. The response speed was measured in two parts. When the light illumination was turned on and off, the voltage was defined as the rising time (τ_r_) and falling time (τ_f_), respectively, at time intervals between 10 and 90% of the output of the normalized voltage. The τ_r_ and τ_f_ are determined as 2.4 and 13.6 ms, respectively. The voltage–time (V–T) characteristic of the device with various frequencies of input light signal is shown in Fig. 4c. A periodic photomodulation was clearly observed with the periodic light signal from 10 to 100 Hz. This demonstrates that the device can operate in a frequency range that is similar to the human eye. Also, the speed of this reaction indicates that the device is capable of detecting a frequency that are difficult for the human eye to detect in the on–off cycle of light illumination.

**Figure 5:**
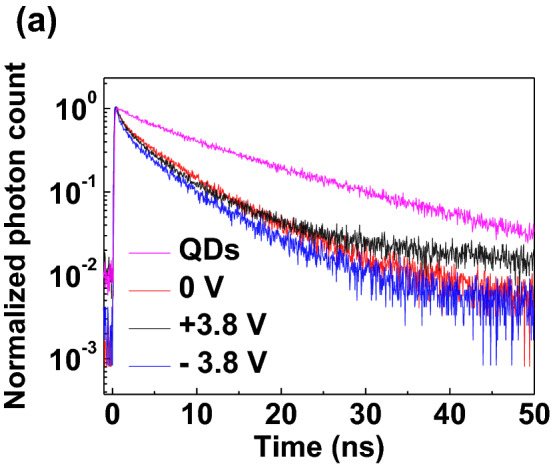
(**a**) Time-resolved photoluminescence decay results of QDs on quartz and full devices with zero bias, light-emitting mode (+ 3.8 V) and light-detecting mode (− 3.8 V), respectively.

Time-resolved photoluminescence (TRPL) measurements using a time-correlated single-photon counting technique were performed to analyze charge decay dynamics as shown in Fig. 5a. A single layer of CdSe/ZnS QDs thin film on quartz and full dual-functional device were prepared for the measurements. The TRPL of the full device was measured at a forward bias of 3.8 V, which gives a sufficiently bright light in the LED mode, and at a reverse bias of − 3.8 V, which is a photodetection mode. To clarify the comparison of the device's charge transition mechanism, measurement at zero bias was performed as well, where the device does not work. During the measurement, the sample was excited using a 400 nm wavelength pulsed laser, and TRPL decay profiles were recorded with a probe at 520 nm wavelength. The PL decay dynamics of the CdSe/ZnS QDs monolayer and full device at various biases were significantly different as shown in Fig. 5a.

The average transient PL decay time <τ> is estimated by fitting the component exponential decay model.

$$\mathrm{D}\left(\mathrm{t}\right)\approx {\sum }_{i=\mathrm{1,2},\dots }{a}_{i}\mathrm{exp}\left(-\frac{t}{{\tau }_{i}}\right)$$

$$<\uptau >=\frac{{a}_{1}{\tau }_{1}^{2}+{a}_{2}{\tau }_{2}^{2}+\cdots }{{a}_{1}{r}_{1}+{a}_{2}{r}_{2}+\cdots }$$

where a_i_ = 1, 2,… is the weighting coefficient, and τ_i_ = 1, 2,… is the PL decay characteristic times. Here, we only use up to i = 2 of the measured values for reasonable calculation (Table 1). The decay curve is applied with a biexponential (i = 1, 2) that includes non- radiative decay (i = 1) and radiative decay (i = 2) values. Radiative decay occurs when photons are absorbed, whereas non-radiative decay occurs due to other factors such as quenching and vibration that do not involve absorption. The PL decay of the full device is faster than that of the CdSe/ZnS QDs film. Therefore, it can be said that the exciton by the light in the QDs of the full device was transferred easily to the other layers. Compared to the PL decay of the full device with 0 V, the PL decay of the device with the forward bias is slower. This means efficient recombination due to reduced exciton transition. And the PL decay with the reverse bias (− 3.8 V) is the fastest, that means the photon energy can transfer to the other charge transport layer efficiently for the light detecting of dual-functional QLEDs. The charge transfer rate constant *κ*_*ct*_ can be estimated from the decay time using the following equation,

**Table 1:** Summary of the average lifetime of charge carriers from the QDs on quartz and full devices with zero bias, light-emitting mode (+ 3.8 V) and light-detecting mode (− 3.8 V).

	${a}_{1}$	${t}_{1}$(ns)	${a}_{2}$	${t}_{2}$ (ns)	Average lifetime <$\tau$> (ns)
QDs	0.422	5.710	0.558	17.072	14.692
0 V	0.422	1.020	0.578	6.743	6.173
+ 3.8 V	0.768	2.138	0.232	13.140	9.288
− 3.8 V	0.571	0.957	0.429	6.576	5.664

$${\kappa }_{ct}=\frac{1}{{\tau }_{1}}+\frac{1}{{\tau }_{2}}$$

where τ_1_ and τ_2_ are the decay characteristic times. Table 2 shows that the electron transition rate is increased at full device compared to QDs on quartz. It is attributed to the conduction level of the other surrounding layers helps to accelerate the transfer of excited charge from QDs by reducing the energy level offset. Besides, it shows a very different electron transition rate results of 0.543 ns^−1^ at + 3.8 V and 1.197 ns^−1^ at − 3.8 V, respectively. In QLEDs mode, excited photons are recombined while not being transferred to the interfacial layer by forward bias, resulting in a low transition rate. In contrast, PD mode excited photons are accelerated to the electrode by reverse bias with the built-in electric field in the surrounding layers. This indicates a high transition rate by inhibiting recombination of excitons.

**Table 2:** Summary of the charge transition rate estimated from the decay time of the QDs on quartz and full devices with zero bias, light-emitting mode (+ 3.8 V) and light-detecting mode (− 3.8 V).

	1/${t}_{1}$ (ns^−1^)	1/${t}_{2} ($ ns^−1^)	Transition rate <$t$^−1^ > (ns^−1^)
QDs	0.175	0.058	0.233
0 V	0.980	0.148	1.128
+ 3.8 V	0.467	0.076	0.543
− 3.8 V	1.044	0.152	1.197

**Figure 6:**
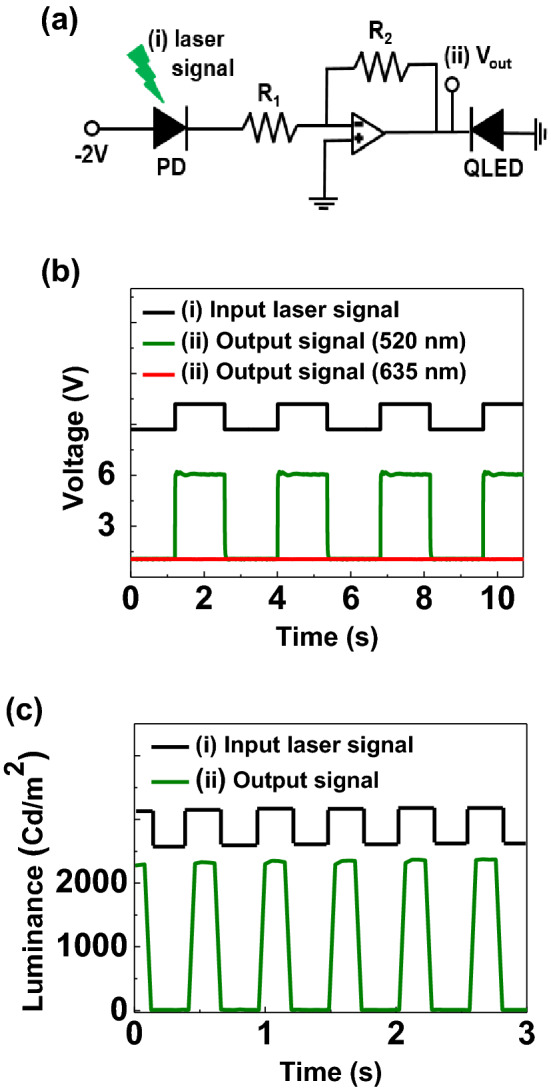
(**a**) Circuit diagram that used two dual-functional QLEDs. Input laser signal enables to produce enough electrical signal from the first device (PD) to turn-on the second device (QLEDs) for the light emission. (**b**) Periodic photoresponses of the PD according to the 520 and 635 nm wavelength light signal measured at (ii) V_out_ of the circuit diagram. (**c**) Luminescence characteristics of the QLEDs according to the periodic input laser signal (λ = 520 nm) into the PD.

A circuit, which can detect a visible light and generate a visible light through two identical dual-functional QLEDs was demonstrated as shown in Fig. 6a. The QLEDs and photodiodes used 5 wt% V_2_O_5_ of dual-functional diodes. The first device (PD) was reverse biased to detect the input 520 nm wavelength light signal, and generated an electrical signal to drive the amplification circuit. Then, the second device (QLEDs), which is forward biased, can emitted a 520 nm wavelength light from the amplified electrical signal, which was generated from the PD. For the amplification of the photocurrent, operation amplifier (Op amp) circuit was used. Figure 6b shows the periodic photoresponses, measured at the position of (ii), just before input the second device. The periodic input laser signal is shown as a black line in the Fig. 6b. When a 520 nm wavelength laser light was used for the input signal, periodic photoresponses was clearly observed. However, it is hard to observe any photoresponses with the illumination of 635 nm wavelength light signal due to the band gap of QDs. Figure 6c shows the periodic emitting light signal from the QLEDs due to the input light signal at the position of (i) on the circuit. Periodic light emitting modulation was clearly observed as shown in the Fig. 6c. Therefore, we can generate a visible light emission from the dual-functional QLEDs by an electrical signal from the visible light signal as shown in the Figure S8, which was generated from the identical device with the reverse bias.

**Figure 7:**
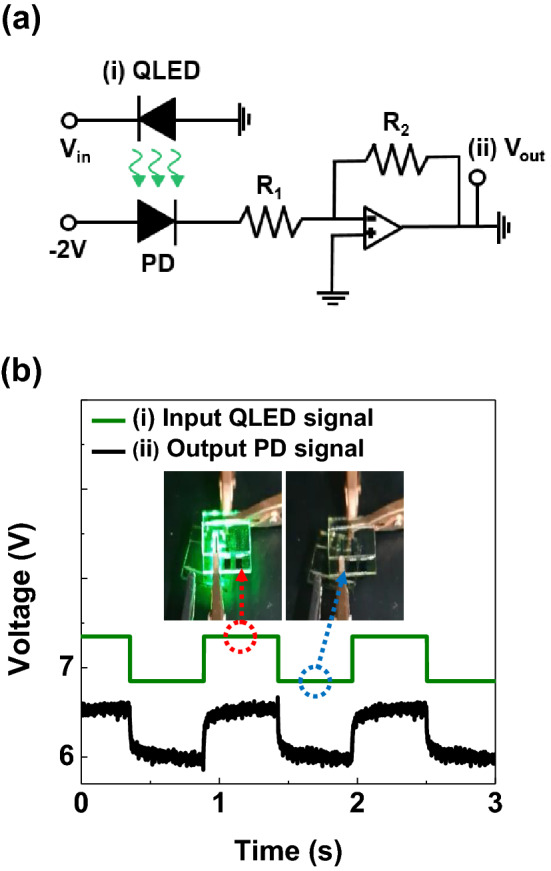
(**a**) Circuit diagram to generate an electrical signal using PD from the light emission of QLEDs. (**b**) Periodic output electrical signal of PD measured at (ii) V_out_ from the input light generated from the (i) dual-functional QLEDs. Inset shows the image of turn-on state QLEDs to generate electrical signal from the PD and turn-off state QLEDs to turn-off the PD.

Figure 7 shows that the light signal from the QLEDs can generate an electrical signal via the other dual-functional device, which was used as a light detection mode. Figure 7a shows the circuit diagram consists of two functional diodes at position. Here in, to detect with high photoresponsivity from a photodiode, the circuit was optimized using QLEDs fabricated with 1 wt% V_2_O_5_. And the photodetector used a 5 wt% V_2_O_5_. A periodic input electrical signal was applied to the first QLEDs to generate a periodic light signal of 520 nm wavelength. The periodic light emitting signal is shown as a green line in Fig. 7b. The periodic light signal was illuminated on the second dual-functional QLEDs to generate an electrical signal. By amplifying the electrical signal using OP amp circuit, a periodic electrical signal was clearly observed at the position of (ii) in the circuit. Inset shows the image of turn-on state and turn-off state of the QLEDs in Fig. 7b. Therefore, dual-functional QLEDs can be used simultaneously as a light emitter and receiver with an appropriate applied bias and enable the developments of efficient visible communications.

## Conclusions

We have fabricated dual-functional QLEDs using a solution processable V_2_O_5_ HIL. The device shows a selectable functionalities of photo-detection and light-emitting via an optimization of V_2_O_5_ (5 wt%) HIL with an appropriate applying voltage. From the measurement of XPS and UPS, we found a gap state in V_2_O_5_ HIL, and the gap state level moved closer to the E_F_ and the DOS of gap state increased, according to increase the concentration of V_2_O_5_ precursor. The change of gap state enables to fabricate a dual-functional QLEDs. The device emitted a bright green light at the wavelength of 520 nm, and with the maximum luminance of 31,668 cd/m^2^ in a forward bias of 8.6 V. Meanwhile, the device could operate as a photodetector in a reverse bias condition. The device was perfectly turned off in a reverse bias with 5 wt% V_2_O_5_ HIL, while an increase of photocurrent was observed during the illumination of a visible-light on the device. Therefore, dual-functional QLEDs can be used simultaneously as a light emitter and receiver with an appropriate applied bias. The result suggests that QLEDs can be used as a photosensor and as a light-emitting diode for the optoelectronics for the next-generation IoT and visible communications.

## Methods

### Materials

Patterned indium-tin-oxide (ITO) glass was purchased from AMG tech, and aluminum (Al) was purchased from Taewon scientific. Vanadium (V) triisopropoxide oxide and poly [(9,9-dioctylfluorenyl-2,7-diyl)-co-(4,4′-(4-s-butylphenyl) diphenylamine)]] (TFB) was purchased from Alfa Aesar and Lumtec, respectively. CdSe/ZnS QDs were purchased from Uniam, and ZnO was purchased from Avantama. p-xylene was purchased from Sigma Aldrich.

### Synthesis of V_2_O_5_ and TFB

Various concentrations (1, 3, and 5 wt%) of the vanadium oxide solution were synthesized by adding vanadium (V) triisopropoxide oxide (0.05, 0.16, and 0.28 ml) into isopropyl alcohol (7 ml), respectively. The solution was mixed for 10 min at 600 rpm using a stirring bar. Then deionized water (0.1 ml) was added, followed by mixing for 50 min to activate the hydrolysis reaction. 1 wt% of TFB solution was prepared by mixing TFB (0.0173 g) into p-xylene (99%, 2 ml). The TFB solution was stirred for 30 min at 600 rpm with a hot plate temperature of 80 °C.

### Fabrication of dual functional diode

Patterned ITO with a sheet resistance of 15 Ω sq^−1^ on a glass substrate was used as the anode. The ITO was rinsed with acetone, IPA and DI water in the order. The work function and the surface hydrophilicity of ITO were increased by using ultraviolet/ozone treatment. The V_2_O_5_ solution was spin-coated onto the ITO surface at 3000 rpm for 1 min. The coated V_2_O_5_ layer was annealed at 150 °C for 15 min and then kept at room temperature for 10 min. TFB solution was spin-coated at 3000 rpm for 30 s onto the V_2_O_5_ layer. The coated TFB layer was annealed at 180 °C for 30 min. The CdSe/ZnS QDs solution was spin-coated at 1500 rpm for 1 min onto the TFB layer. The coated CdSe/ZnS QDs layer was annealed at 180 °C for 30 min. The ZnO solution was spin-coated at 2000 rpm for 1 min onto the CdSe/ZnS QDs layer. The coated ZnO layer was annealed at 180 °C for 30 min. Al cathode was deposited by using a thermal evaporator with a shadow mask. Finally, the device was encapsulated using a thin glass with an epoxy.

### Characterization

The interfacial electronic structures and chemical states were measured using UPS and XPS with an ultra-high vacuum system (1 $\times$ 10^–9^ Torr). Photoelectron spectra were recorded using a KTRATOS AXIS NOVA system. He-I (21.218 eV) source was used for the UPS measurements, and x-ray source for XPS measurement was Al Kα (1486.6 eV). Energy resolutions were approximately 0.1 eV and 1.0 eV for UPS and XPS, respectively. No sample temperature control was attempted during the experiments. Current–voltage–luminance (I–V–L) characteristics of QLEDs were measured using a conventional measurement system (M6100, McScience). Current–voltage (I–V) characteristics of photodiodes were measured by using a probe station and 4145B semiconductor parameter analyzer. The carrier transfer mechanism was analyzed via time-resolved photoluminescence (TRPL) measurement. The periodic optical response behavior were evaluated using a function generator and an oscilloscope.

## Supplementary Information

Supplementary Tables.
