# Supplementary material for: Dual-functional quantum-dots light emitting diodes based on solution processable vanadium oxide hole injection layer
Source: Sci Rep. 2021 Jan 18;11:1700. doi: 10.1038/s41598-021-81480-5 (PMC7814015; doi:10.1038/s41598-021-81480-5)
Supplement: Supplementary file 1 — Supplementary Tables. [file 41598_2021_81480_MOESM1_ESM.docx]

**Supporting Information**

Dual-functional quantum-dots light emitting diodes based on solution processable vanadium oxide hole injection layer

Tae Yeon Kim^1^, Sung Ho Park^1^, Byung Jun Kim^1^, Su Been Heo^1^, Jong Hun Yu^1^, Jae Seung Shin^1^, Jong-Am Hong^2^, Beom-Su kim^2^, Young Duck Kim^2^, Yongsup Park^2^ and Seong Jun Kang^1^

^1^ Department of Advanced Materials Engineering for Information and Electronics, Kyung Hee University, Yongin 17104, Korea

^2^ Department of Physics and Research Institute for Basic Sciences, Kyung Hee University, Seoul 02447, Korea

**Index**

**Figure S1.** UPS spectra result from the SEC and valence region for ITO, V_2_O_5_, QD, TFB, ZnO and Al, respectively. The inset shows the gap state level of V_2_O_5_ HIL.

**Figure S2.** Tauc’s plots of the (a) V_2_O_5_, (b) TFB, (c) QDs and (d) ZnO films from UV/Vis absorption spectra, where α is the absorption coefficient, hν is the photon energy.

**Figure S3.** High-Resolution TEM image of the device structure with 3 wt% and 5 wt% V_2_O_5_.

**Figure S4.** Current-Voltage (I-V) characteristics in a log plot with 1, 3 and 5 wt% concentration of the V_2_O_5_, respectively.

**Figure S5.** (a) Current density and luminance versus applied voltage curves with 3 wt% and 5 wt% V_2_O_5_ HIL of QLEDs. (J-L-V) (b) Electroluminescence spectra with 3 wt% and 5 wt% V_2_O_5_ HIL of QLEDs. (c) Current efficiency versus voltage curves according to V_2_O_5_ HIL concentrations.

**Figure S6.** (a) Absorbance spectra of active layer QDs.

**Figure S7.** Responsivity at (a) -1V, (b) -2V and (c) -3V in PD mode with illumination wavelength variations.

**Figure S8.** (a) Image that emits a dual functional diode using a photocurrent generated when 520 nm and 635 nm input laser signal are irradiated onto diode at PD mode.

**Table S1.** Table of responsivity for illumination wavelength and voltage variations.


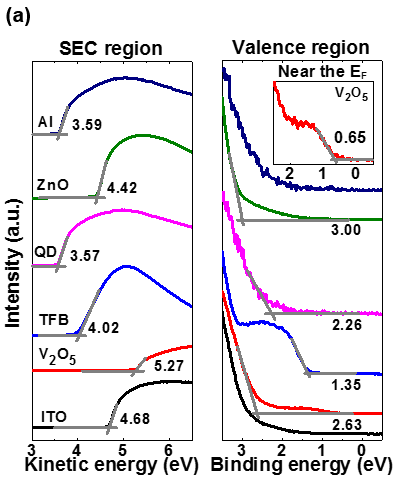


**Figure S1.** UPS spectra result from the SEC and valence region for ITO, V_2_O_5_, QD, TFB, ZnO and Al, respectively. The inset shows the gap state level of V_2_O_5_ HIL.


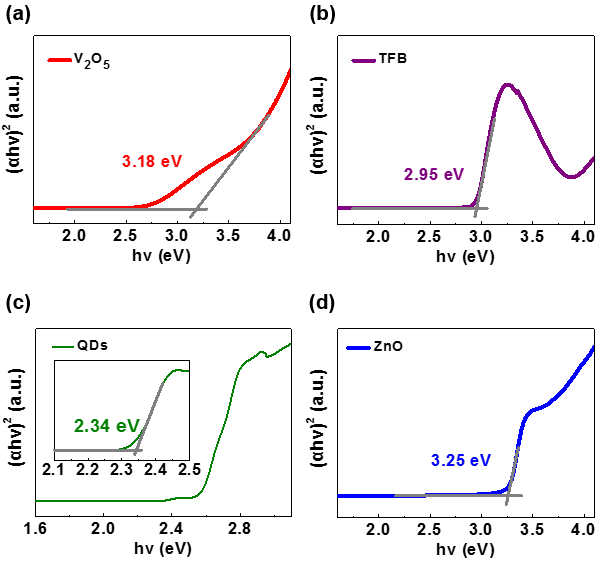


**Figure S2.** Tauc’s plots of the (a) V_2_O_5_, (b) TFB, (c) QDs and (d) ZnO films from UV/Vis absorption spectra, where α is the absorption coefficient, hν is the photon energy.


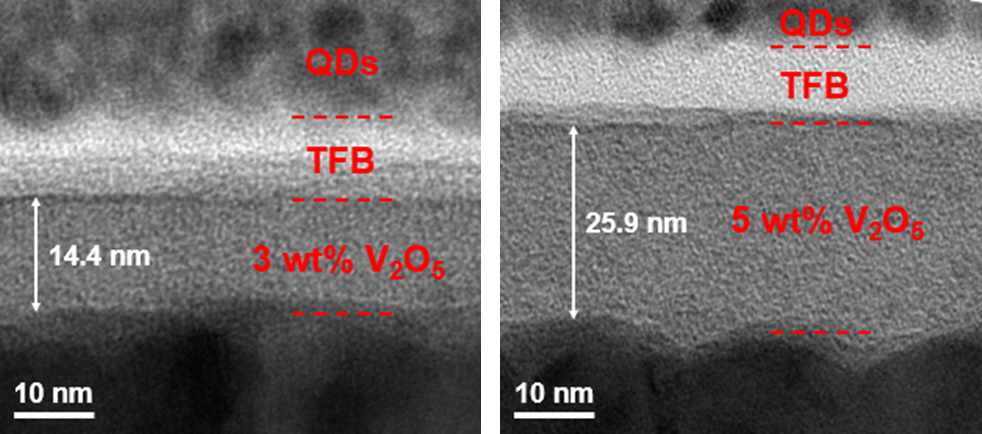


**Figure S3.** High-Resolution TEM image of the device structure with 3 wt% and 5 wt% V_2_O_5_.


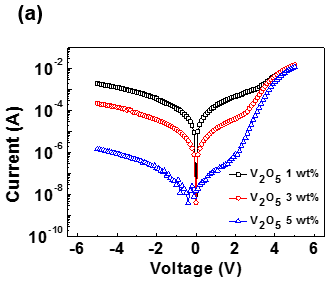


**Figure S4.** Current-Voltage (I-V) characteristics in a log plot with 1, 3 and 5 wt% concentration of the V_2_O_5_, respectively.


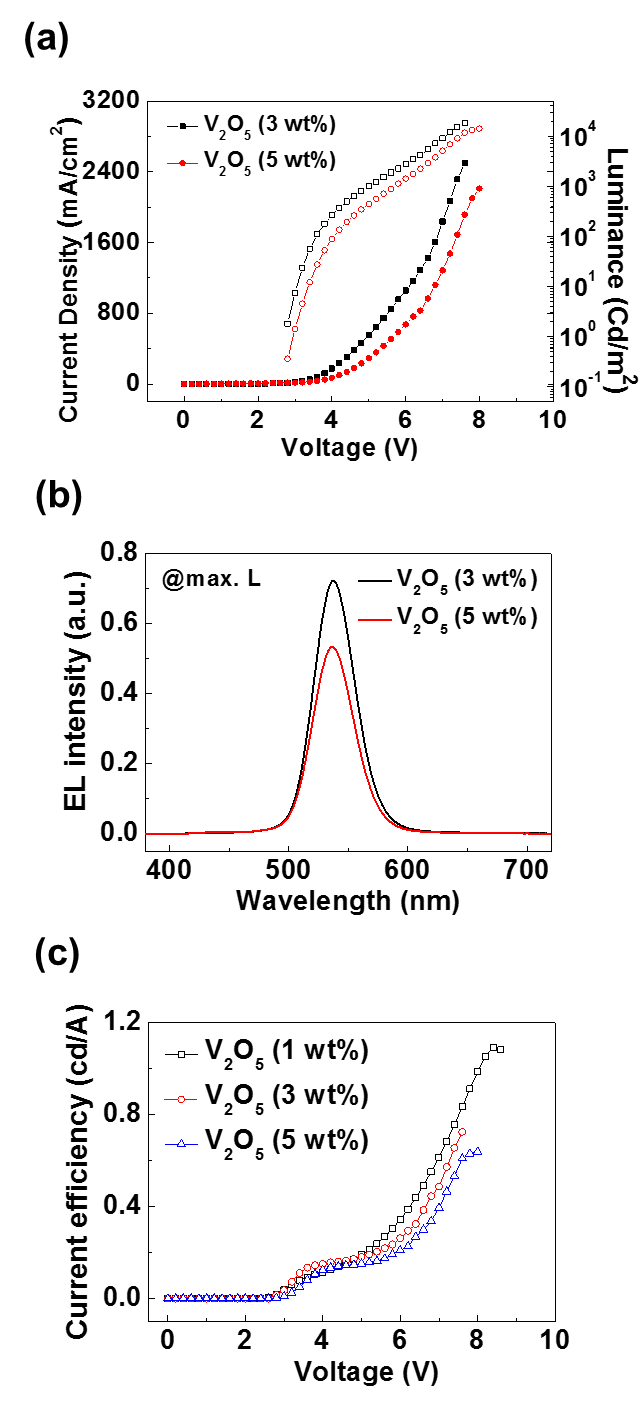


**Figure S5.** (a) Current density and luminance versus applied voltage curves with 3 wt% and 5 wt% V_2_O_5_ HIL of QLEDs. (J-L-V) (b) Electroluminescence spectra with 3 wt% and 5 wt% V_2_O_5_ HIL of QLEDs. (c) Current efficiency versus voltage curves according to V_2_O_5_ HIL concentrations.


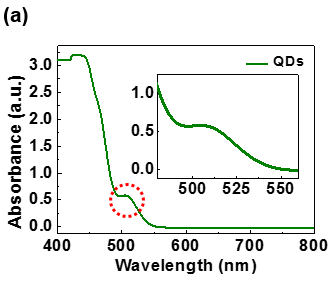


**Figure S6.** (a) Absorbance spectra of active layer QDs.


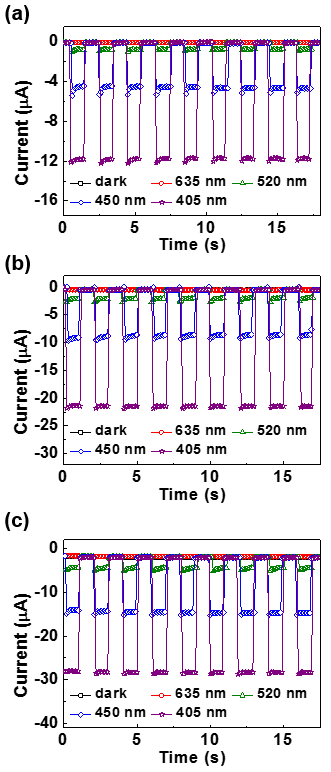


**Figure S7.** Responsivity at (a) -1V, (b) -2V and (c) -3V in PD mode with illumination wavelength variations.


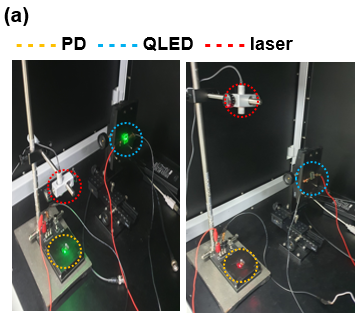


**Figure S8.** (a) Image that emits a dual functional diode using a photocurrent generated when 520 nm and 635 nm input laser signal are irradiated onto diode at PD mode.


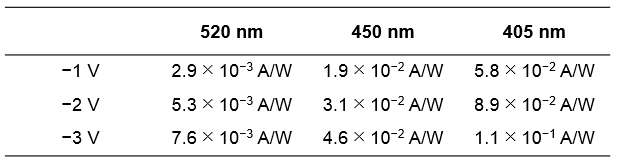


**Table S1.** Table of responsivity for illumination wavelength and voltage variations.
